# The Role of Toll-like Receptors and Viral Infections in the Pathogenesis and Progression of Pulmonary Arterial Hypertension—A Narrative Review

**DOI:** 10.3390/ijms262211143

**Published:** 2025-11-18

**Authors:** Agnieszka Styczeń, Martyna Krysa, Paulina Mertowska, Ewelina Grywalska, Tomasz Urbanowicz, Maciej Krasiński, Malwina Grobelna, Weronika Topyła-Putowska, Mansur Rahnama-Hezavah, Michał Tomaszewski

**Affiliations:** 1 Department of Cardiology, Medical University of Lublin, Jaczewskiego 8, 20-093 Lublin, Poland; styczen.agnieszka@gmail.com (A.S.); m.janczewska2002@gmail.com (M.K.); michal.tomaszewski@umlub.edu.pl (M.T.); 2 Department of Experimental Immunology, Medical University of Lublin, 20-093 Lublin, Poland; ewelina.grywalska@umlub.edu.pl; 3 Cardiac Surgery and Transplantology Department, Poznań University of Medical Sciences, 1/2 Dluga Street, 61-848 Poznań, Poland; turbanowicz@ump.edu.pl; 4 Department of Obstetrics and Gynaecology, St Mary’s Hospital, Imperial College Healthcare Trust, London W2 1NY, UK; 5 Department of Vascular, Endovascular Surgery, Angiology and Phlebology, Poznan University of Medical Science, 1/2 Długa Street, 61-848 Poznań, Poland; 6 Chair and Department of Oral Surgery, Medical University of Lublin, 20-093 Lublin, Poland

**Keywords:** Toll-like receptors, viral infection, pulmonary arterial hypertension, therapeutic target

## Abstract

Aberrant activation of innate immunity promotes the development of pulmonary arterial hypertension (PAH); however, the role of pattern recognition by Toll-like receptors (TLRs) within the pulmonary vasculature remains unclear. To consolidate knowledge (as of June 2025) about TLRs and their interactions with viruses in PAH and to identify therapeutic implications. A narrative review of experimental and clinical studies investigating ten TLRs in the context of the pulmonary vascular microenvironment and viral infections. Activation of TLR1/2, TLR4, TLR5/6, TLR7/8, and TLR9 converges on the MyD88–NF-κB/IL-6 axis, thereby enhancing endothelial-mesenchymal transition, smooth muscle proliferation, oxidative stress, thrombosis, and maladaptive inflammation, ultimately increasing pulmonary vascular resistance. Conversely, TLR3, through TRIF–IFN-I, preserves endothelial integrity and inhibits vascular remodeling; its downregulation correlates with PAH severity, and poly (I:C) restitution has been shown to improve hemodynamics and right ventricular function. HIV-1, EBV, HCV, endogenous retrovirus K, and SARS-CoV-2 infections modulate TLR circuits, either amplifying pro-remodeling cascades or attenuating protective pathways. The “TLR rheostat” is shaped by polymorphisms, ligand biochemistry, compartmentalization, and biomechanical forces. The balance between MyD88-dependent signaling and the TRIF–IFN-I axis determines the trajectory of PAH. Prospective therapeutic strategies may include TLR3 agonists, MyD88/NF-κB inhibitors, modulation of IL-6, and combination approaches integrating antiviral therapy with targeted immunomodulation in a precision approach.

## 1. Introduction

Pulmonary arterial hypertension (PAH) is a rare, progressive disorder characterized by elevated pulmonary arterial pressure and pulmonary vascular resistance, ultimately leading to right ventricular failure and death if left untreated. Despite significant advances in pharmacological therapies targeting endothelial dysfunction, vasoconstriction, and smooth muscle cell proliferation, the pathogenesis of PAH remains incompletely understood, and its prognosis continues to be poor [1,2,3,4].

Recent evidence suggests that chronic inflammation, immune dysregulation, and aberrant innate immune signaling play crucial roles in the initiation and progression of PAH [5,6,7,8]. Among the principal components of the innate immune system, Toll-like receptors (TLRs)—a family of pattern recognition receptors (PRRs)—have emerged as pivotal mediators linking environmental and microbial stimuli to vascular remodeling, endothelial dysfunction, and immune cell infiltration within the pulmonary circulation [9,10,11,12]. TLRs recognize conserved molecular motifs derived from pathogens (pathogen-associated molecular patterns, PAMPs) or endogenous danger signals (damage-associated molecular patterns, DAMPs), thereby initiating downstream signaling cascades that culminate in the production of cytokines, chemokines, and interferons (Table 1) [9,13,14].

In humans, ten TLRs (TLR1–TLR10) are expressed. The role of specific receptors in pulmonary vascular disease remains an active area of investigation. Several preclinical studies have demonstrated the upregulation of TLR2, TLR3, TLR4, and TLR9 in lungs and vascular cells in experimental PAH models [15,16,17,18]. Functionally, TLR activation contributes to the core pathological mechanisms of PAH—including endothelial cell apoptosis, proliferation of pulmonary arterial smooth muscle cells (PASMCs), oxidative stress, and sustained inflammation—thereby driving vascular remodeling and hemodynamic deterioration [6,19,20]. In contrast, certain receptors, most notably TLR3, exert protective effects by modulating interferon signaling and preserving endothelial integrity, underscoring the receptor-specific balance within TLR networks that may ultimately shape disease trajectory and therapeutic opportunities [21,22].

**Table 1:** Summary of ligands recognized by Toll-like receptors (TLR1–TLR10): pathogen-associated molecular patterns (PAMPs) and endogenously derived danger signals (DAMPs) and their significance in immunopathology [23,24,25,26,27,28,29,30,31,32,33,34].

TLR	PAMP	DAMP
TLR1/2	Triacylated bacterial lipopeptides (e.g., Pam3CSK4; TLR1/2 heterodimer).	No specific, reliable DAMPs for TLR1 itself; DAMP signaling usually via TLR2-dependent heterodimers.
TLR2	Gram(+) bacteria: lipopeptides, lipoteichoic acid, peptidoglycan; zymosan (fungi); often as TLR2/6.	HMGB1, hyaluronan fragments, biglycan, HSP;
TLR3	dsRNA viral; agonist: poly(I:C).	Endogenous dsRNA/exosomal RNA from necrotic cancer cells/tissues.
TLR4	LPS (Gram−); classic endotoxin receptor.	HMGB1, S100A8/A9, heme, ECM fragments (e.g., hyaluronan), and fibronectin-EDA are the TLRs with the most significant number of proposed DAMPs (some of which are still under discussion).
TLR5	Bacterial flagellin.	There are no widely accepted endogenous DAMPs for humans.
TLR6/2	Diacylated bacterial lipopeptides (e.g., Pam2CSK4; TLR2/6 heterodimer).	As for TLR2, HMGB1/ECM/HSP has been reported, but data for TLR6 alone are sparse.
TLR7	viral ssRNA (GU-rich); agonists: imidazoquinolines.	Endogenous miRNAs (e.g., miR-21, let-7, miR-154-5p); RNA–LL37 complexes facilitating self-RNA recognition.
TLR8	Viral ssRNA; R848 and other small molecules.	Extracellular miRNAs (e.g., miR-21/miR-29a in tumor exosomes); RNA–LL37 complexes activating TLR8 in neutrophils.
TLR9	Unmethylated CpG bacterial/viral DNA; agonist: CpG ODN.	mtDNA (also oxidized) and DNA–LL37 complexes facilitating the recognition of one’s own DNA.
TLR10	Function/ligands remain ambiguous; lipopeptides are suggested (often as TLR2/10); the role is more likely to be modulatory/inhibitory.	No reliably confirmed DAMPs; receptor still considered “orphan”.

Abbreviations: TLR—Toll-like receptor, PAMP—Pathogen-associated molecular pattern, DAMP—Danger-associated molecular pattern, HMGB1—High mobility group box one protein, HSP—Heat shock protein, dsRNA—Double-stranded RNA, poly(I:C)—Polyinosinic: polycytidylic acid (synthetic dsRNA analog), LPS—Lipopolysaccharide, S100A8/A9—S100 calcium-binding proteins A8 and A9 (also known as calprotectin), ECM—Extracellular matrix, ssRNA—Single-stranded RNA, miRNA (miR)—MicroRNA, LL37—Human cathelicidin antimicrobial peptide (LL-37), R848—Resiquimod (synthetic imidazoquinoline, TLR7/8 agonist), CpG ODN—CpG oligodeoxynucleotide (synthetic unmethylated CpG DNA), mtDNA—Mitochondrial DNA.

Furthermore, the interaction between TLR activation and viral infections has garnered attention as a potential trigger in the development of PAH, particularly among predisposed individuals. Human viruses such as HIV, Epstein–Barr virus, and SARS-CoV-2 can enhance pulmonary vascular remodeling and immune activation through direct and indirect mechanisms involving TLR pathways [17,34,35,36,37,38]. In predisposed hosts (e.g., with polymorphisms in TLR/adapter genes, attenuated IFN-I response, autoimmune diseases, or environmental exposures), viral PAMPs (ssRNA/dsRNA, CpG DNA, envelope proteins) and secondary DAMPs released from damaged tissues (mtDNA, HMGB1, NETs) activate TLR2/4 on endothelium and smooth muscle cells, as well as TLR3/7/8/9 on antigen-presenting cells and adventitia. This results in the activation of the MyD88–NF-κB/IL-6 axis and, in context, the TRIF–IFN-I axis, which promotes endothelial barrier dysfunction, endothelial-mesenchymal transition, PASMC proliferation, fibrotic remodeling, thromboinflammatory microangiopathy, and sustained leukocyte recruitment. Additionally, virally derived or host miRNAs carried in extracellular vesicles can allosterically amplify TLR7/8/9 signaling. The reactivation of latent viruses (e.g., EBV) under conditions of chronic inflammation can act as a secondary trigger, sustaining a pathological positive feedback loop. Accumulating evidence indicates that the TLR–virus axis represents an important, modifiable hub in PAH pathobiology, which may account for the heterogeneity of clinical phenotypes and differences in treatment response [17,34,35,36,37,38].

In this narrative review, we aim to synthesize current knowledge on the role of Toll-like receptors in the pathogenesis and progression of pulmonary arterial hypertension. We further highlight the contribution of viral infections to TLR-mediated immune responses in PAH, with particular emphasis on the translational relevance of TLRs as potential biomarkers and therapeutic targets.

## 2. The Role of Toll-like Receptors in the Pathogenesis and Progression of Pulmonary Arterial Hypertension

### 2.1. TLR1 and TLR2

TLR1 and TLR2 constitute the functional TLR1/2/6/10 subfamily encoded on chromosome 4, in which heterodimerization-dependent signaling at the plasma membrane enables recognition of a broad spectrum of microbial lipopeptides [39,40]. TLR1 (CD281) is a 786-amino acid transmembrane glycoprotein containing 19 LRR repeats, abundantly expressed in the spleen, kidney, and lung of adults and—at the cellular level—on B and T lymphocytes (CD4+), monocytes/macrophages, selected dendritic cell subpopulations, astrocytes, microglia, and platelets; relatively lower expression is observed in the fetal brain and liver [17,41,42,43]. This receptor preferentially binds triacylated bacterial lipopeptides and some parasitic proteins, in the absence of reliably confirmed endogenous ligands; after ligand binding, it most often acts as a TLR1/2 heterodimer, initiating the MyD88→NF-κB cascade and inducing proinflammatory cytokines. In the context of barrier physiology, TLR1 is involved in sensing the gut microbiota and recognizing *Borrelia burgdorferi* surface lipoproteins, which determines an effective response to the etiological agent of Lyme disease [44,45]. TLR2 (CD282), which shares a similar structure (784 amino acids, 19 LRRs), is present on B and T lymphocytes—including Tregs (CD4+CD25+)—neutrophils, monocytes/macrophages, mast cells, myeloid dendritic cells, Schwann cells, and microglia. It recognizes complex bacterial lipo- and glycopeptides, the lipoteichoic acid of Gram-positive bacteria, and fungal β-glucans. In addition, Hsp70, gp96, and signals from necrotic cells have been described as potential DAMPs [46,47,48,49]. Functionally, TLR2 acts cooperatively with other receptors: in association with TLR1, it preferentially recognizes triacylated lipopeptides, whereas with TLR6, it preferentially detects diacylated lipopetides. In both cases, receptor activation leads to the recruitment of MyD88, subsequent activation of NF-κB, and the expression of inflammatory mediators, forming a key module of innate pathogen recognition and integration of environmental signals into the effector response [46,47,48,49].

In a rat model of monocrotaline-induced pulmonary hypertension (MCT-PAH), macrophage infiltration in the lung tissue was observed, accompanied by impaired necroptosis dependent on receptor-interacting protein kinase-3 (RIPK3) and activation of the associated TLR pathway [50]. In 2023, Lian et al. attempted to better explain the role of single Toll-like receptors in the development and progression of PAH induced in Sprague-Dawley rats. The animals were administered monocrotaline subcutaneously twice at an interval of 7 days, at a dose adjusted to the animal’s body weight (20 mg/kg b.w.). The study examined the characteristics of infiltrating cells in the immune system and sought potential biomarkers for PAH using a comprehensive bioinformatics approach. Critical central genes were identified. The bioinformatics tool CIBERSORT was employed to assess the characteristics of infiltrating immune cells. Subsequently, the correlation between central genes and immune cells was examined using Pearson analysis [51].

Lian et al. demonstrated that the expression of *TLR1* genes in rats with MT-PAH was reduced in comparison to the control group. Moreover, this expression correlated positively with the number of neutrophils and monocytes. On the other hand, a negative correlation was observed between TLR1 gene expression and the number of resting CD4+ T lymphocytes. This test confirmed that the TLR1 receptor plays a pivotal role in the functioning of the innate immune system. It is also worth noting that, as a representative of the TLR family, it participates in the recognition of a wide range of pathogens. The positive correlation between *TLR1* expression and the number of monocytes and macrophages suggests that TLR1 may contribute to the development of PAH by regulating neutrophil- and monocyte-related immune responses, as well as the progression of subsequent inflammatory states [51] (Table 2).

Overall, TLR2 plays a significant role in the pathogenesis of pulmonary arterial hypertension by participating in the activation of endothelial cells and vascular smooth muscle cells, thereby contributing to vascular remodeling, and facilitating the development of the inflammatory response. Preclinical studies have shown that TLR2 activation is associated with reduced apoptosis and proliferation of PASMC, increased production of growth factors (e.g., TGF-beta, PDGF), as well as the induction of fibroblasts and extracellular matrix deposition. Excessive activation of TLR2 in animal models leads to oxidative stress, increased expression of adhesion molecules (ICAM-1, VCAM-1)—which facilitates leukocyte migration—and endothelial dysfunction (impaired permeability and impaired nitric oxide production). Nevertheless, it is essential to emphasize that the most crucial pathophysiological mechanism by which TLR2 activation contributes to the development of PAH is the induction of chronic inflammation. The induction occurs via the activation of the NF-kB pathway and the production of proinflammatory cytokines, including IL-1, IL-6, and TNF-alpha, as accompanied by the recruitment of macrophages and neutrophils, and the ability to maintain the already initiated chronic inflammation in the pulmonary vessels. Furthermore, enhanced TLR2 activity correlates with the severity of the clinical condition and with the deterioration of the right ventricular function of the animals. Therefore, modifying the activity of this receptor may in the future become a potential therapeutic target in the treatment of PAH [15,42,52,53] (Table 2).

Notably, Broen et al. demonstrated that a rare polymorphism in the *TLR2* gene is associated with the systemic sclerosis phenotype and enhances the production of inflammatory mediators. The described trial revealed that among patients with systemic sclerosis, the rare TLR2 Pro631His variant is associated with antitopoisomerase positivity (the SSc diffuse manifestation) and the development of PAH. Additionally, this variant has an impact on TLR-2-mediated cellular responses. Specifically, monocyte-derived dendritic cells carrying the Pro63His variant cause the excessive production of proinflammatory cytokines (IL-5 and NRF-alpha), which is a consequence of TLR2 activation. However, more research is required on this topic to clarify the significance of TLR2 in the pathogenesis of both SSc and PAH [43].

### 2.2. TLR3

**Figure 1:**
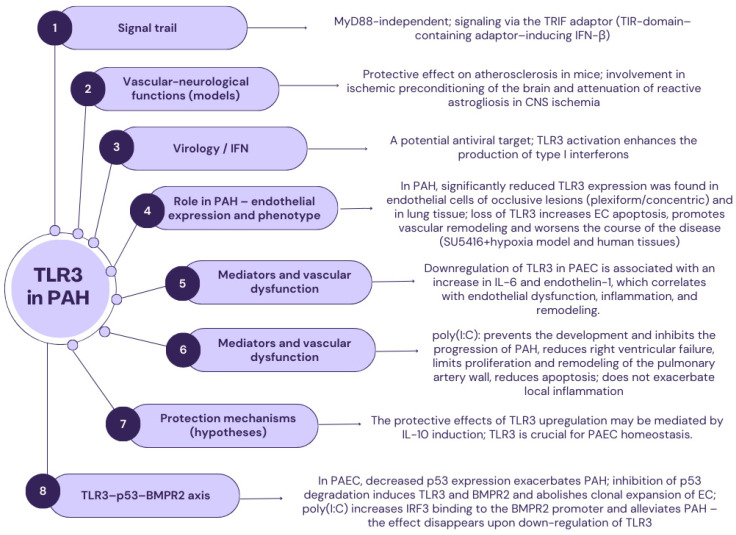
TLR3 in PAH—signaling, vascular-neurological functions, endothelial phenotype, mediators, interventions, and the TLR3–p53–BMPR2 axis (based on [21,22,28,54,55,56,57,58,59,60,61,62,63,64,65,66,67]). Abbreviations: TLR3—Toll-like receptor 3, MyD88—Myeloid differentiation primary response 88, MyD88-independent—Signaling that does not require MyD88, TRIF—TIR-domain–containing adaptor-inducing interferon-β, TIR—Toll/Interleukin-1 receptor (domain), IFN-β—Interferon beta, IFN—interferons, CNS—Central nervous system, PAH—Pulmonary arterial hypertension, EC—Endothelial cell(s), PAEC—Pulmonary artery endothelial cell(s), IL-6—Interleukin-6, poly(I:C)—Polyinosinic: polycytidylic acid (synthetic dsRNA analog; TLR3 agonist, BMPR2—Bone morphogenetic protein receptor type 2, p53—Tumor protein p53, IRF3—Interferon regulatory factor 3, SU5416—Vascular endothelial growth factor receptor (VEGFR) inhibitor used to induce experimental PAH.

TLR3, a transmembrane glycoprotein also known as CD283, consists of 904 amino acids and contains 23 LRRs. The genes encoding *TLR3* are located on chromosome 4. This receptor is located intracellularly in various cell types, including B lymphocytes and dendritic cells. The intracellular organelles in which the cell membrane TLR3 is expressed are the endoplasmic reticulum, phagosomes, and early endosomes. Exogenous ligands for TLR3 are viral or synthetic double-stranded RNA and polycytidylic acid, whereas mRNA constitutes the TLR3 endogenous ligand [54,55]. TLR3 is a unique receptor in the entire family of TLR due to its activation occurring independently of the MyD88 protein (Figure 1). The further signaling cascade uses the adaptor protein TIR-domain-containing adapter-inducing interferon-β (TRIF). In the division of all TLR3 into subfamilies, this receptor constitutes a separate subfamily on its own [56,57,58,59].

In the human body, the highest expression of TLR3 is found in the cells of the pancreas and the placenta. In relation to the cardiovascular system, activators of TLR3 have been shown to affect human vascular cells [54,55]. It has been observed that TLR3 plays a protective role against the development of atherosclerotic lesions in a mouse model of the disease [60,61]. Additionally, stimulation of TLR3 signaling is associated with ischemic preconditioning-induced protection against brain ischemia. It has also been demonstrated that the TLR is responsible for the attenuation of reactive astrogliosis, a phenomenon observed in cases of central nervous system ischemia [62,63,64]. TLR3 is also a promising target for antiviral drugs, as its activation in the final phase of the immune response leads to the production of substantial amounts of interferon-1 subtypes [28,65,66].

Evidence shows that TLR3 plays a protective role in the pathogenesis of pulmonary arterial hypertension. In a 2019 clinical study, Farkas et al. hypothesized that the expression of *TLR3* is an essential process for maintaining endothelial cell homeostasis and protection, and the activation of TLR3 with drugs is a potential therapeutic target in the treatment of pulmonary arterial hypertension [21]. The study was conducted using a mouse model of pulmonary arterial hypertension induced by chronic hypoxia and the active substance SU5416, and also examined fragments of human lung tissue. It demonstrated a significantly reduced expression of the TLR3 receptor in the endothelial cells of vascular occlusive changes, typical of PAH. Moreover, decreased expression of PAH in the lung tissue of animals was observed. This phenomenon resulted in a marked exacerbation of the disease and enhanced endothelial cell apoptosis in mice exposed to chronic hypoxia and treated with the SU5416 substance. In addition, it was found that in the endothelium of plexiform and concentric vascular lesions, there was a significant loss of *TLR3* expression. At the same time, in the control group of patients, endothelial cells showed strong expression of this receptor. It should also be emphasized that the study confirmed reduced expression of *TLR3* in endothelial cells in cultured pulmonary artery cells (PAEC) and in lung tissue with induced pulmonary arterial hypertension. These findings led to the conclusion that the loss of TLR3 expression leads to pulmonary vascular remodeling, resulting in a more intensive vascular remodeling process [21].

The next stage of the study assessed the intensity of the caspase-dependent apoptosis process of pulmonary artery endothelium cells. The study group demonstrated an increased number of apoptotic PAEC intimal cells, accompanied by reduced *TLR3* expression. The increased intensity of the apoptosis process in cells characterized by reduced *TLR3* expression was also confirmed in vitro. It is worth noting that the study also showed that reduced *TLR3* expression results in increased expression of genes for IL-6 and endothelin-1, which consequently promotes a gene expression profile that favors increased vascular remodeling, endothelial dysfunction, and the development of local inflammation [21].

Farkas D. et al. also examined the effect of the dsRNA substance PolyC (I:C), a TLR3 agonist, on the functioning of PAEC. It has been demonstrated that treatment with this substance prevents the development of PAH, inhibits its progression, and reduces the severity of right ventricular failure. Moreover, it diminishes the intensity of proliferation and remodeling of the pulmonary artery wall, while simultaneously limiting its apoptosis. Furthermore, the administration of Poly (I:C) did not intensify local inflammation. In summary, the study demonstrated for the first time that reduced *TLR3* expression is present in the lung tissue and endothelial cells of patients with PAH, both in vitro and in vivo, which leads to increased cell susceptibility to apoptosis and the development of PAH. Moreover, both the dsRNA of the substance PolyC (I:C)—polyinosinic/polycytidylic acid and the TLR3 agonist increase *TLR3* gene expression and reduce the severity of existing PAH. This protective effect of TLR3 up-regulation is most likely mediated by the induction of interleukin-10. Based on the entire study, a key conclusion was drawn that the expression of TLR3 is a crucial element in maintaining PAEC homeostasis. Therefore, it is necessary to conduct further research to identify active substances that restore appropriate TLR3 expression, which may represent a new and effective therapeutic strategy for treating PAH, complementing existing methods [21,67].

In 2023, Bhagwani A.R. et al. attempted to supplement the existing knowledge about TLR3 in the pathogenesis of PAH. The researchers aimed to elucidate the roles of the TLR3 receptor and p53 protein in the clonal expansion and pathogenesis of PAH by regulating bone morphogenetic protein type 2 (BMPR2) signaling. The study demonstrated reduced gene expression of the p53 protein in PAEC, which exacerbated existing pulmonary arterial hypertension. Additionally, it was observed that reducing p53 degradation resulted in the induction of TLR3 and BMPR2 and the abolition of clonal expansion of EC, thus alleviating the course of PAH. In addition, the substance Poly(I:C) was responsible for the increased binding of interferon regulatory factor (IRF3) to the BMPR2 promoter, thereby reducing the severity of pulmonary arterial hypertension in mice with activated p53 protein. This phenomenon was not observed in animals with TLR3 downregulation [22].

### 2.3. TLR4

**Figure 2:**
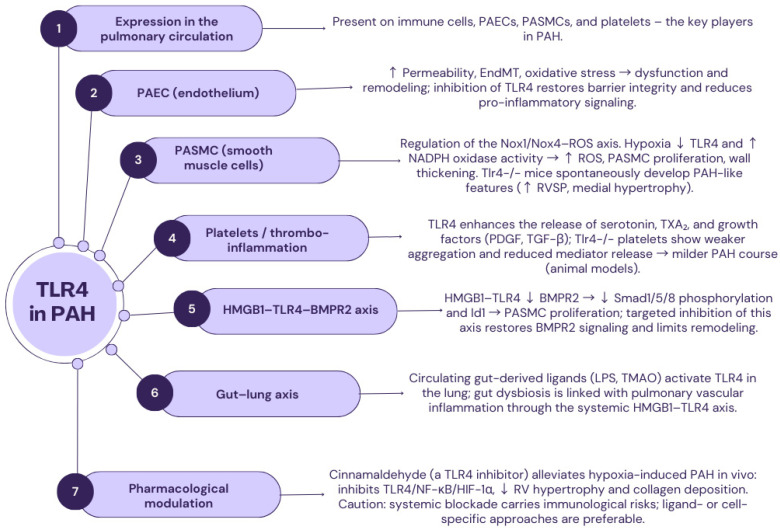
Key aspects of TLR4 involvement in pulmonary arterial hypertension (PAH)—based on expression, signaling mechanisms, cellular targets, vascular consequences, and translational implications (based on [24,69,70,71,72,73,74,75,76,77,78,79,80,81,82,83,84,85,86,87,88,89,90,91,92,93,94,95]). Abbreviations: PAH—Pulmonary arterial hypertension, PAEC—Pulmonary artery endothelial cells, PASMC—Pulmonary artery smooth muscle cells, LPS—Lipopolysaccharide, HMGB1—High mobility group box 1 protein, NF-κB—Nuclear factor kappa-light-chain-enhancer of activated B cells, EndMT—Endothelial-to-mesenchymal transition, ROS—Reactive oxygen species, Nox1/Nox4—NADPH oxidase isoforms 1 and 4, NADPH oxidase—Nicotinamide adenine dinucleotide phosphate oxidase, TXA_2_—Thromboxane A_2_, PDGF—Platelet-derived growth factor, TGF-β—Transforming growth factor beta, BMPR2—Bone morphogenetic protein receptor type 2, Smad1/5/8—SMAD family transcription factors 1, 5, and 8, Id1—Inhibitor of DNA binding 1, TMAO—Trimethylamine-N-oxide, HIF-1α—Hypoxia-inducible factor 1-alpha, ↑ means increase, ↓ means decrease, → denotes a relationship, a causal connection, or a transition to the next piece of information.

The genes encoding *TLR4*, also known as CD284, are located on chromosome 9, and the entire receptor is composed of 839 amino acids [68]. At the histological level, TLR4 is situated outside the cell on various cell types, including neutrophils, T lymphocytes, monocytes/macrophages, mast cells, myeloid dendritic cells, intestinal epithelial cells, and breast cancer cells [69,70,71]. Ligands for this particular transmembrane glycoprotein include many chemical substances, the most important of which, and simultaneously the only mentioned exogenous ligand, is lipopolysaccharide, which is found in all Gram-negative bacteria and some Gram-positive bacteria. Endogenous ligands for TLR4 include fragments of heparan sulfate, hyaluronic acid, fibrinogen, various opioid drugs, heat shock proteins, and nickel [72,73,74]. Among all TLRs, TLR4 constitutes a distinct subfamily. This receptor is also an exception among all receptors, as it is the only one that can transmit a signal in the pathway using the MyD88 protein, both with and without its participation (Figure 2) [69,70,75].

In the human body, TLR4 plays a pivotal role due to its proven involvement in the pathogenesis of numerous infectious and neoplastic diseases. It has been demonstrated that stimulation of TLR4 with lipopolysaccharide or its natural derivatives activates its robust anti-cancer activity through the production of pro-inflammatory cytokines and type 1 interferon [76,77].

TLR4 has also been shown to have a significant role in the occurrence of long-term adverse effects of opioid drugs. Activation of TLR4 results in the release of substances that modulate the current inflammatory state, including IL-1β and TNF-α. The constant presence of these substances in the human body most likely causes a decrease in the effectiveness of opioid treatment after some time. This, in turn, leads to the development of tolerance, allodynia, and hyperalgesia [78,79]. For a long time, scientific studies have also highlighted the role of TLR4 in both the pathogenesis and development of neurodegenerative diseases, such as Parkinson’s disease, Alzheimer’s disease, and Huntington’s disease [80,81,82].

TLR4 is a critical mediator of innate immunity and vascular homeostasis. Its dysregulation has been increasingly implicated in the pathogenesis of pulmonary arterial hypertension, where chronic inflammation, vascular remodeling, and thrombosis play key roles. In the pulmonary circulation, TLR4 is expressed not only on immune cells but also on endothelial cells, pulmonary artery smooth muscle cells (PASMCs), and platelets—key cellular players in the development of PAH [19,69,72,83,84,85,86,87].

Activation of TLR4 by exogenous ligands (e.g., lipopolysaccharide) or DAMPs such as high-mobility group box one protein (HMGB1), fibronectin, or hyaluronic acid, triggers signaling cascades via MyD88 and TRIF, converging on NF-κB, AP-1, and interferon regulatory factors, and inducing pro-inflammatory cytokines such as IL-6 and TNF-α [24,88].

In pulmonary arterial endothelial cells (PAECs), TLR4 activation promotes increased permeability, endothelial-to-mesenchymal transition (EndMT), and oxidative stress, leading to vascular dysfunction and remodeling. Inhibition of TLR4 in experimental models has been shown to restore endothelial barrier integrity and reduce pro-inflammatory signaling [24,88].

In PASMCs, TLR4 regulates redox balance via the Nox1/Nox4–ROS axis. Hypoxic conditions suppress TLR4 expression and enhance NADPH oxidase activity, promoting ROS generation, PASMC proliferation, and vessel wall thickening. TLR4-deficient mice spontaneously develop PAH-like features, including increased right ventricular systolic pressure (RVSP) and medial hypertrophy of pulmonary arteries [87,89].

Platelet-expressed TLR4 contributes to thromboinflammation by enhancing the release of vasoactive substances, including serotonin, thromboxane A2, and growth factors (e.g., PDGF, TGF-β). TLR4-deficient platelets exhibit attenuated aggregation and reduced release of inflammatory mediators, resulting in milder PAH severity in animal models [90,91].

HMGB1–TLR4 signaling has also been shown to suppress bone morphogenetic protein receptor 2 (BMPR2) expression, a hallmark of PAH pathophysiology. This inhibition reduces Smad1/5/8 phosphorylation and Id1 expression, promoting PASMC proliferation. Targeted inhibition of the HMGB1–TLR4 axis restores BMPR2 signaling and attenuates pulmonary vascular remodeling [92,93].

Furthermore, gut-derived ligands such as LPS and trimethylamine-N-oxide (TMAO) can activate TLR4 in the lung, linking gut dysbiosis to pulmonary vascular inflammation via the systemic HMGB1–TLR4 axis [94,95].

Pharmacologic modulation of TLR4 offers promise. Cinnamaldehyde, a natural TLR4 inhibitor, alleviates hypoxia-induced PAH in vivo by suppressing the TLR4/NF-κB/HIF-1α pathway, reducing right ventricular hypertrophy and collagen deposition [52,89]. However, systemic TLR4 blockade may carry immunological risks; future strategies should prioritize ligand- or cell-specific targeting.

### 2.4. TLR5 and TLR6

TLR5 (CD285) is a receptor whose coding genes are located on chromosome 1. Its exact location in human body cells has not been determined so far. However, it has been demonstrated that TLR5 is located on the outer surface of cells, including B lymphocytes, regulatory lymphocytes, macrophages, and dendritic cells. TLR5 consists of 20 LRRs, and the entire receptor is composed of 858 amino acids. TLR5 is a separate subfamily among all TLRs [96]. Activation of TLR5 occurs through the MyD88 protein pathway, and the pathogen-associated molecular pattern for this described transmembrane glycoprotein is flagellin, a component of the flagella of Gram-negative bacteria, which serves as an extrinsic ligand for the receptor. The endogenous ligands for TLR5 are still unknown [97,98,99,100]. It has also been demonstrated that aberrant functioning of TLR5 is associated with the onset of inflammatory diseases, including inflammatory bowel disease and rheumatoid arthritis, as well as with the development of neoplastic diseases, such as breast, cervical, endometrial, gastric, and ovarian cancer [101,102,103,104,105,106,107].

The TLR5-Nox4 signaling pathway has been proven to be a key element in the development of atherosclerosis. It has been observed that the flagellin-TLR-5-Nox4 axis stimulates the movement of vascular smooth muscle cells and, consequently, initiates the formation of atherosclerotic plaque. It was shown that the flagellin-TLR5-Nox4 axis enhances inflammatory processes in the endothelium by activating the NF-kB factor, inducing the expression of proinflammatory cytokines such as IL-6, IL-8, RANTES (Regulated on Activation, Normal T-cell Expressed and Secreted), and the adhesion factor ICAM-1 (intercellular adhesion molecule-1). It was also noted that in mice lacking Nox4, both of the processes mentioned above were significantly less intense [108,109,110,111,112].

To date, there are no dedicated functional studies in PAH models that precisely determine the role of TLR5 in the pathogenesis and progression of this disease (Table 3). Transcriptomic analysis (using a mouse model with pulmonary hypertension) showed a significant induction of *TLR5* expression in the lung tissue of patients with PAH. TLR5 recognizes flagellin in bacteria, activating the MyD88 →IRAK→NF-kB pathway, which leads to the release of cytokines such as TNF-alpha, IL-6, and IL-8. On this basis, it can be assumed that it thereby stimulates the formation of a local inflammatory state, analogously to TLR2 and TLR4, which are responsible for the recruitment of leukocytes, oxidative stress, and the phenomenon of vascular remodeling. As TLR5 may play a pro-inflammatory and remodulatory role, further studies, both in vivo and in vitro, should be conducted to assess the potential for intervention in PAH therapy by modulating TLR5 function [113,114,115].

The genes encoding *TLR6* are located on chromosome 4. In the human body, TLR6 (CD286) is situated extracellularly in cells such as B lymphocytes, monocytes/macrophages, and mast cells. This receptor consists of 19 LRRs and 796 amino acids. TLR6, similarly to TLR5, is activated via the MyD88 protein pathway, which stimulates the nuclear factor NF-kB. The exogenous ligands for TLR6 are multiple diacyl lipopeptides, while endogenous ligands for TLR6 have not been discovered yet. TLR6 belongs to the TLR1/2/6/10 subfamily of receptors [116,117].

It has been proven that A 359T>C single-nucleotide polymorphism in the structure of the extracellular LRR of TLR6 predisposes to Legionnaires’ Disease development [118]. Moreover, there are assumptions that the Ser249Pro polymorphism in TLR may be associated with a greater incidence of asthma bronchialis in some populations [119].

Grote K. et al. attempted to determine the role of this transmembrane glycoprotein in the functioning of the vascular system. They conducted studies showing a significant participation of the TLR2/TLR6 complex in the process of angiogenesis. It was observed that lipopolysaccharide, a macrophage-activating lipopeptide of 2 kDa (MALP-2), commonly found in bacteria, stimulated the formation of new vessels through a pathway mediated by the TLR2/TLR6 complex, the activation of which causes the release of granulocyte-macrophage colony-stimulating factor (GM-CSF). Stimulation of angiogenesis, involving the described complex, was confirmed to occur both in vitro and in vivo [120].

No scientific papers have been published to explain the importance of TLR6 in PAH in direct clinical trials. Only transcriptional RNA-seq analyses are available in an animal model of PAH induced, for example, by monocrotaline. These studies demonstrate that stimulation of the TLR6/TLR2 heterodimer in PAMSCs by typical bacterial ligands activates the NF-κB pathway, promoting the production of IL-1β, IL-6, and TNF-α, which are crucially involved in pulmonary vascular remodeling. Although there are no direct studies describing TLR6 activity in animals with PAH, there are strong indirect indications that activation of this receptor promotes vascular proliferation and remodeling, the increase of endothelin-1 levels, and the development of local inflammation in PAH. Due to the dynamic growth of knowledge on PAH, further research is also necessary in this field to evaluate TLR6 as a potential therapeutic target in PAH, which could be achieved by antagonizing signals in the TLR2/TLR6 heterodimer and NF-κB axis (Table 3) [15,42].

### 2.5. TLR7 and TLR8

**Figure 3:**
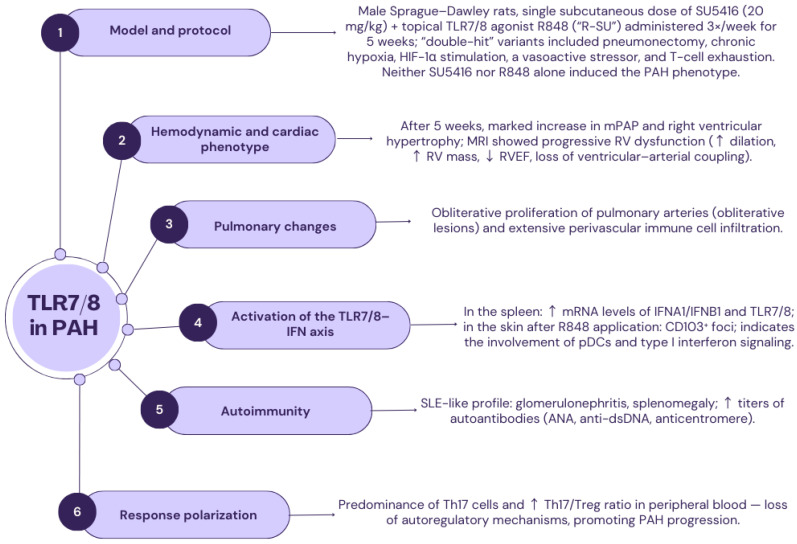
Key Observations on TLR7/8 Activation in Experimental Pulmonary Arterial Hypertension (PAH) (based on [121,122,123,124,125,126,127,128,129,130,131,132,133,134,135,136,137,138,139,140,141,142]). Abbreviations: PAH—Pulmonary arterial hypertension, SU5416—Vascular endothelial growth factor receptor (VEGFR) inhibitor used to induce experimental PAH, s.c.—Subcutaneous, R848 (R-SU)—Resiquimod, synthetic imidazoquinoline and TLR7/8 agonist, HIF-1α—Hypoxia-inducible factor 1-alpha, mPAP—Mean pulmonary arterial pressure, RV—Right ventricle, MRI—Magnetic resonance imaging, RVEF—Right ventricular ejection fraction, IFNA1—Interferon alpha 1, IFNB1—Interferon beta 1, pDC—Plasmacytoid dendritic cells, IFN—Interferon, SLE—Systemic lupus erythematosus, ANA—Antinuclear antibodies, anti-dsDNA—Anti–double-stranded DNA antibodies, Th17—T helper 17 cells, Treg—Regulatory T cells, ↑ means increase; ↓ means decrease.

The genes *TLR7* and *TLR8*, which are subsequent transmembrane glycoproteins, are located on the X chromosome in the human body. TLR7 (CD287) is located intracellularly in structures such as lysosomes, endoplasmic reticulum, and phagosomes; however, the precise location of TLR8 (CD288) inside the cell remains unknown [121,122]. Both TLR7 and TLR8 are composed of 25 LRRs. Both receptors have a similar number of amino acids—TLR consists of 1049 amino acids, while TLR8 is composed of 1041 amino acids. TLR7 and TLR8, together with TLR9, form one of the six subfamilies of TLRs, distinguished by amino acid sequence homology. The single-stranded DNA of viruses constitutes an extrinsic ligand for both TLR7 and TLR8, while their intrinsic ligands have not been identified so far. Activation of the signaling cascade of TLR7 and TLR8 receptors occurs using the MyD88 protein [121,122,123,124,125]. TLR7 is present on B lymphocytes and plasmacytoid dendritic cells, and when activated, it produces signaling using interferon-gamma. In contrast, TLR8 induces NF-κB-dependent cytokines in myeloid cells (Figure 3) [126,127,128,129,130,131].

In the human vascular system, TLR7 and TLR8 act as “guardians” of immunity, selectively recognizing subsets of RNA sequences crucial for an effective response against viruses [132,133]. It has also been proven that constant activation of TLR7/8 results in excessive production of proinflammatory signals, often pathological, as exemplified by a vast “cytokine storm” in the course of viral pneumonia, usually leading to the patients’ death. It has been noted that the abnormal production of interferon, mediated by TLR9 activation, can trigger a reaction exhibiting the characteristics of an autoimmune relationship, as evidenced by the repolarization of T helper lymphocytes and the autoreactive expansion of B lymphocytes [127,134]. Moreover, the local application of the TLR7/TLR8 receptor agonist resiquimod (R848) has been observed to lead to the development of autoimmune disorders similar to those observed in systemic lupus erythematosus, which is characterized by excessive production of pro-inflammatory cytokines, the production of autoantibodies, and multi-organ damage [134,135].

Yeh F.-C. et al. conducted a study using rats in 2023 to clarify the role of autoimmune phenomena induced by stimulation of TLR7 and 8 in the perpetuation of pulmonary artery endothelial damage and vascular dysfunction caused by the VEGF antagonist Sugen 5416 (SU5416) in the initial stages and subsequent stages of progression of pulmonary vascular disease in rats [136]. As is well known, SU5416 is a factor that induces endothelial cell apoptosis—a transient but specific pathophysiology underlying the phenomenon of pulmonary vascular remodeling. In the present study, SU5416 was used in combination with other factors that could potentially influence the exacerbation of pulmonary artery remodeling, such as pneumonectomy, chronic hypoxia, stimulation with hypoxia-inducible factor-1α, the presence of a vasoactive stressor, and the phenomenon of T cell exhaustion. All the described phenomena were aimed at generating experimental PAH in vivo [136].

The study used male rats weighing between 180 g and 200 g. The animals were given a single subcutaneous injection of SU5416, adjusting the dose of the substance to the animal’s body weight (20 mg/kg b.w.). Then, the rats were administered the TLR7/8 agonist R840 (R-SU) externally three times a week for a total of 5 weeks. After 5 weeks, the animals developed pulmonary hypertension with a phenotype associated with a severe course, including a significant increase in mPAP and significant right ventricular hypertrophy. In histological preparations of the lung tissue of 5W R-SU rats, obstructive proliferation of pulmonary vessels was found. Furthermore, histologically, extensive perivascular infiltration of immune cells was observed in the examined tissue. Neither SU5416 nor R848 was able to induce the pathomechanisms responsible for the development of PAH. Infiltrations of immune cells were only evident in rodents exposed to two substances simultaneously, using the ‘double hit’ principle, located around vascular changes in the lungs, as well as in the enlarged lymphoid tissue associated with the bronchi (Figure 3) [135].

Immunohistochemical preparations revealed a focal accumulation of CD103+ cells at the epidermal-dermal junction of the ears of rats treated externally with R848. Secondly, the animals showed increased expression of mRNA for interferons *IFNA1* and *IFNB1*, as well as mRNA for *TLR7* and *TLR8* in the spleen, indicating the involvement of pDCs (perivascular dendritic cells) due to the activation of TLR7 and TLR8. It is believed that autoimmune processes stimulated by TLR7 activity demonstrate the action of SU5416-sustaining destructive stimuli for pulmonary blood vessels, which ultimately lead to vascular remodeling of an obliterative nature [135,136].

The study also included a group of rats that were given a prolonged 7-week exposure to SU5416, after which heart of the animals was imaged using MRI (BioSpec 9.4 T) at fixed time points—after 2, after 5, and at the time point after 7 weeks (maintained for another 2 weeks without R848). It is worth emphasizing that in rats, the extended 7-week control point protocol revealed proliferation of pulmonary arteries and increased mPAP at a similar level to the group of animals subjected to a 5-week exposure to SU5416. Data obtained from MRI studies were analyzed, noting a successive decrease in right ventricular efficiency and progression of PAH, which was expressed by progressive dilatation of the right ventricular cavity and hypertrophy of the right ventricular muscle, as well as by impairment of right ventricular function (e.g., reduced right ventricular ejection fraction—RVEF) and loss of ventricular-arterial coupling [135].

In R-SU rats, autoimmune reactions analogous to those observed in patients diagnosed with SLE were observed, with involvement of numerous internal organs, including glomerulonephritis and splenomegaly [137]. A significantly increased expression of IFNA1/IFNAB1 and TLR7 and TLR8 was observed in the spleen cells of R-SU rats, which is evidence of the animals’ response to substance R848 [136]. Repolarization towards Th17 lymphocytes was observed in the peripheral blood of the studied animals, characterized by an increased ratio of Th17 to regulatory T lymphocytes. This phenomenon is attributed to the activation of TLR7 and TLR8 and is an expression of the loss of autoregulatory mechanisms and the development of autoimmune reactions. They have been described in relation to the degree of severity and prognosis in different PAH groups [137,138,139]. It is worth emphasizing that significantly increased titers of autoantibodies were detected in the plasma of R-SU rats, including antinuclear antibodies, antibodies against double-stranded DNA, and anticentromere antibodies. Autoantibody production is a common immunopathological mechanism observed in patients with PAH. The study results also emphasized that activation of TLR7/8 receptors leads to fatal complications of SLE, such as malignant arterial hypertension and systemic vasculitis in female rats, in which the observed greater susceptibility to the development of autoimmune diseases induced by TLR7 activation may be related to the dose effect—the gene expression of the biallelic X chromosome [140,141,142].

### 2.6. TLR9

TLR9 (CD289), composed of 1032 amino acids, is a receptor located on chromosome 3. In its structure, TLR9 contains 25 tandem leucine-rich repeats. Histologically, TLR9 is situated inside the cell, in structures such as lysosomes, endoplasmic reticulum, and phagosomes [143]. The exogenous ligands for TLR9 are bacterial and viral CpG DNAs, whereas the internal ligands are antibody-chromatin complexes. The mechanism of action of the described transmembrane glycoprotein consists of detecting molecules associated with damage to the body’s own tissues (DAMPs), which come from inside the cell [144,145]. Although the role of Toll-like receptors in the pathogenesis of cardiovascular diseases is the subject of numerous scientific studies, it remains largely unexplained. Several years ago, it was discovered that tissue injury inducing the release of nuclear or mitochondrial CpG (5′—C—phosphate—G—3′) DNA can stimulate the TLR9 receptor as a DAMP [146,147,148,149,150,151,152,153,154,155,156,157,158]. Moreover, it has been suggested that Toll-like receptor 9, by sensing mitochondrial DNA that has been previously excised and transported to the cytosol, may play a “gatekeeper” function at the border between the vascular system and the immune system [149].

Recent studies have highlighted the importance of mitochondrial DNA production and subsequent inflammation, moderated by TLR9, as key factors in the pathogenesis of various cardiovascular diseases [150,151,152,153]. It has also been proven that episodes of acute left ventricular heart failure, caused by TLR9-mediated inflammatory reactions due to the release of mtDNA (mitochondrial DNA) damaged due to hemodynamic stress, have been observed in mice with transverse aortic stenosis [153]. It has been noted that circulating damaged mtDNA activates TLR9 and is partially responsible for the increase in blood pressure in rats with spontaneous hypertension [152]. More recent studies also emphasize the role of TLR9 activation and subsequent stimulation of IL-6 as a proinflammatory cytokine in the migration and proliferation of smooth muscle cells in the endothelium of pulmonary arteries in rats [154].

The TLR9 receptor has been identified as a pro-inflammatory receptor in relation to the pathogenesis of PAH, as it is responsible for activating NF-κB, IL-6, and subsequent inflammatory processes in animal models. In a 2021 study by Tomohito Ishikawa et al., it was hypothesized that mtDNA could activate TLR9, thereby stimulating the accumulation of inflammatory cells and IL-6 production in the lungs of rats, leading to the progression of pulmonary arterial hypertension. The study was conducted on 165 adult male rats (Sprague Dawley) weighing between 200 and 250 g, in which pulmonary arterial hypertension was induced with a single subcutaneous injection of monocrotaline adjusted for the animal’s body weight (60 mg/kg b.w.). Three types of study protocols were used: a preventive protocol, a short-term reversal protocol, and an extended reversal protocol. The tested rats were divided into two groups: one randomly administered a selective TLR9 antagonist, E6446 (provided by Eisai Inc., São Paulo, Brazil), and the other a non-selective quinoline agonist, most closely related to TLR9 among all Toll-like receptors, chloroquine (Sigma-Aldrich, Saint Louis, MO, USA). The study then assessed the effect of TLR9 activation on the value of hemodynamic parameters in the pulmonary circulation, vascular remodeling, and survival of rats [155]. In rats administered monocrotaline, a significant increase in the concentration of mtDNA markers in blood serum, activation of TLR9, and increased levels of *IL-6* mRNA in lung tissue were observed approximately 14 days after injection of the active substance. Moreover, animals exposed to monocrotaline exhibited altered hemodynamic parameters in the pulmonary circulation. On the 21st day after the administration of the alkaloid, an increase in the value of the right ventricular systolic pressure, increased total peripheral resistance, and more intensive remodeling of pulmonary vessels, together with the activation of macrophages, were observed [155].

In the preventive protocol, administration (from −3 to 21 days after monocrotaline injection) of a selective or nonselective TLR9 inhibitor significantly reduced the increase in right ventricular systolic pressure and total pulmonary vascular resistance index, as well as vascular remodeling, and macrophage accumulation at day 21. These inhibitors also significantly reduced NF-κB activation and *IL-6* mRNA levels to a similar extent. In the short-term reversal protocol, E6446 treatment (days 14–17 after monocrotaline injection) virtually normalized NF-κB activation and *IL-6* mRNA levels and reduced macrophage accumulation. In an extended reversal protocol, E6446 treatment (days 14–24 after monocrotaline injection) reversed total pulmonary vascular resistance index and vascular remodeling and further increased survival rates in monocrotaline-exposed rats [155].

**Figure 4:**
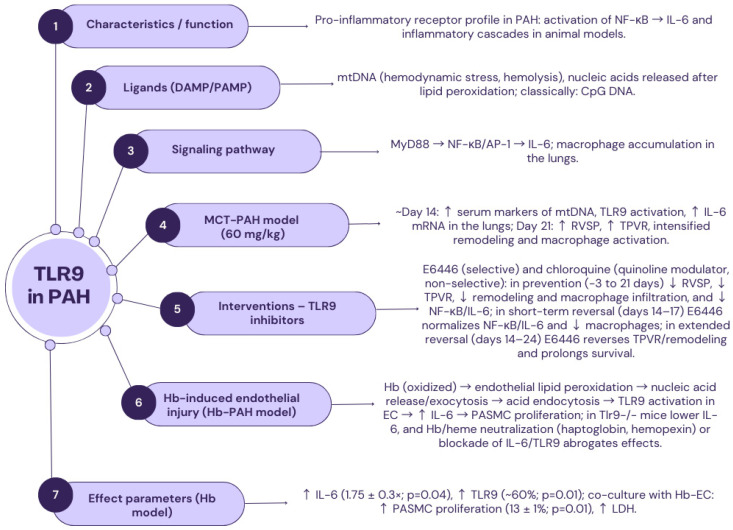
TLR9 in pulmonary arterial hypertension (PAH). (based on [144,145,146,147,148,149,150,151,152,153,154,155,156,157,158,159,160,161,162,163,164,165,166,167]). Abbreviations: PAH—Pulmonary arterial hypertension, NF-κB—Nuclear factor kappa-light-chain-enhancer of activated B cells, IL-6—Interleukin-6, DAMP—Danger-associated molecular pattern, PAMP—Pathogen-associated molecular pattern, mtDNA—Mitochondrial DNA, CpG DNA—Cytosine-phosphate-guanine DNA motifs (unmethylated bacterial/viral DNA), MyD88—Myeloid differentiation primary response 88, AP-1—Activator protein 1, MCT-PAH—Monocrotaline-induced pulmonary arterial hypertension, RVSP—Right ventricular systolic pressure, TPVR—Total pulmonary vascular resistance, E6446—Selective TLR9 antagonist, Hb—Hemoglobin, EC—Endothelial cells, PASMC—Pulmonary artery smooth muscle cells, LDH—Lactate dehydrogenase, ↑ means increase, ↓ means decrease, → denotes a relationship, a causal connection, or a transition to the next piece of information.

After completing the study, Ishikawa et al. formulated three essential conclusions. Firstly, activation of TLR9 participates in the pathogenesis of monocrotaline-induced pulmonary arterial hypertension in rats via activation of the NF-κB-IL-6 pathway. Secondly, long-term inhibition of TLR9 activity not only impedes progression but also reverses the pathophysiological phenomena observed in the lungs and reduces the expression of mRNA of the main proinflammatory interleukin in the pathogenesis of PAH, i.e., IL-6. Thirdly, reduced TLR9 activity leads to prolonged survival in rats exposed to the alkaloid described [81]. During the trial, it was also noted that vascular stress plays a key role in the pathogenesis and maintenance of perivascular inflammation, which is dependent on NF-κB-IL-6, as well as in the phenomenon of vascular remodeling in rats with PAH. In conclusion, the inhibition of TLR9 activity is a promising therapeutic target for the future in the treatment of this chronically progressive, incurable disease. However, the extrapolation of the studies conducted in the rat model to the human model of pulmonary arterial hypertension remains somewhat controversial, and further studies are needed to clarify the way in which hemodynamic stress induces mtDNA production in the lungs, and to determine the exact cell type stimulating TLR9 activation (Figure 4) [155,156].

The role of endothelial TLR9 in the development of PAH induced by hemoglobin (Hb)-induced tissue injury has also been studied [155]. It is widely believed that pulmonary vascular inflammation plays a crucial role in mediating the development and progression of PAH-related hemolytic anemia. However, the mechanisms by which hemoglobin located in the extracellular compartment promotes vascular inflammation, thereby leading to PAH, have not yet been fully explained. It is hypothesized that free cellular Hb may influence the progression of PAH due to its vigorous chemical reaction with NO, which results in reduced NO bioavailability and pulmonary artery vasoconstriction [157,158,159,160]. In clinical conditions, the release of hemoglobin as a result of hemolysis may occur in various diseases, including severe sepsis, hemolytic anemia [160,161,162,163], extracorporeal membrane oxygenation (ECMO), and chronic renal replacement therapy (CRRT) [161]. It is worth emphasizing that excessive amounts of released hemoglobin and peroxides stimulate the local microenvironment, which is rich in DAMPs, thereby activating TLRs and potentially initiating or exacerbating the progression and clinical symptoms of PAH [162,163].

Loomis et al. [154] attempted to prove the hypothesis that hemoglobin-induced lipid peroxidation and subsequent endothelial cell injury are associated with the activation of TLR9 located in the endothelium, leading to IL-6-mediated proliferation of smooth muscle cells in the pulmonary arteries. The research group consisted of male and female mice weighing between 20 and 25 g, which were subjected to jugular vein cannulation and then underwent surgery. The test assessed the effect of hemoglobin on endothelial cells (mPAEC) and smooth muscle cells (mPASMC) of the pulmonary arteries of the studied animals. Substances inhibiting TLR9 and IL-6 were then used, as well as Hb and heme-binding proteins, i.e., haptoglobin and hemopexin. The role of Hb in vivo was also examined using myeloid differentiation cells typical of the primary response 88 and mice that do not express TLR9. In animals, IL-6 concentration was measured in peripheral blood serum, *TLR9* expression was assessed based on the mRNA concentration for this receptor in plasma, and systolic pressure in the right heart chamber was measured by direct puncture of this cavity with the chest closed [154].

The study showed increased expression of IL-6 (1.75 ± 0.3-fold; *p* = 0.04) and a significant increase in TLR9 expression (60%; *p* = 0.01). In contrast, in mice lacking TLR9, a lower level of IL-6 expression was noted in the lung tissue compared to wild-type cohorts. It was also demonstrated that Hb in its oxidized form is responsible for inducing lipid peroxidation. In mice co-cultured with activated hemoglobin endothelium, significantly higher concentrations of nucleic acids, more intense multiplication of pulmonary artery smooth muscle cells, and significantly higher concentrations of lactate dehydrogenase in blood serum were observed. The study proved that the proliferative activity of mouse PASMC was increased (13 ± 1%; *p* = 0.01) during co-culture with activated Hb mPAEC (Figure 4) [154].

Summarizing, Hb stimulates lipid peroxidation of cell membranes, which may be the initial step in a locally limited inflammatory response mediated by TLR9, resulting in the presentation of an activated PASMC phenotype and ultimately leading to the development of pulmonary arterial hypertension. The process of lipid peroxidation leads to the release of nucleic acids by cell death or their excision from damaged mitochondrial DNA. Then, the phenomenon of acid endocytosis occurs, which activates TLR9 on adjacent endothelial cells. This leads to the upregulation of IL-6, promoting the proliferation and growth of SMC with a phenotype characteristic of pulmonary vascular disease. The toxicity of Hb and heme is attributed to their prooxidant properties, which cause endothelial cell damage and apoptosis by inducing the peroxidation process [154,164,165,166,167].

### 2.7. TLR10

TLR10 (CD290) is the least described receptor in medical literature in relation to all 10 receptors found in humans. It is known that chromosome 4 is the site where the genes encoding *TLR10* are situated. This transmembrane glycoprotein is composed of 811 amino acids and 19 LRRs. Histologically, TLR10 is located outside the cell; however, its precise location remains a subject of research. It is most likely present on B lymphocytes, monocytes/macrophages, and intestinal epithelial cells. Based on amino acid homology, TLR10 belongs to the TLR2/TLR6/TLR10 subfamily of receptors. The exogenous ligands for TLR10 are triacylated lipopeptides, and induction of signal transduction from TLR10 occurs via the MyD88 protein. The endogenous ligands for TLR10 remain unknown [168,169,170,171,172,173,174,175,176]. Recently, it has been discovered that other potential ligands for TLR10 may include *Borrelia burgdorferi*, *Listeria monocytogenes*, and *Helicobacter pylori* LPS (TLR2/10), as well as HIV-1 gp41 and H1N1/H5N1 [177,178,179].

There are currently no scientific studies available on the potential involvement of TLR10 in cardiovascular diseases and its role in the pathogenesis of PAH. Hypothetically, since activation of the TLR10 receptor has anti-inflammatory effects, this receptor could play a protective role in the vascular system. Activation of TLR10 could therefore result in reduced endothelial cell damage, reduced proliferation, and reduced local inflammation in the vessels. Further clinical trials are undeniably needed in this area.

## 3. The Role of Viral Infections in the Development and Progression of Pulmonary Arterial Hypertension

Viral infections are increasingly recognized as a “second hit” that initiates or perpetuates cascades leading to PAH. In genetically or environmentally predisposed individuals, infections—including HIV-1, EBV, HCV, SARS-CoV-2, and reactivation of endogenous retroviruses—can affect the pulmonary vascular wall both directly and indirectly. Direct effects include dysfunction and apoptosis of endothelial cells (PAEC), endothelial-to-mesenchymal transition (EndMT), and smooth muscle cell remodeling (PASMC). Indirectly, viral PAMPs and secondary DAMPs (e.g., mtDNA, HMGB1) activate Toll-like receptors and their adaptors, switching the response from the protective TRIF-IFN-I axis (typical of TLR3) to the proinflammatory MyD88-NF-κB/IL-6 pathways—including TLR2/4/7/8/9. This results in persistent inflammation, oxidative stress, immunothrombosis, and disruption of the BMPR2-Smad axis, which together drive vascular remodeling and increased pulmonary resistance. Viral infections also modulate adaptive immunity, promoting Th17 polarization, T cell exhaustion, the activation of plasmacytoid dendritic cells (pDCs), and type I interferon production, as well as—in some phenotypes—the development of autoantibodies. Preclinical data suggest that TLR3 agonism—e.g., poly(I:C)—may be protective, whereas TLR7/8/9 activation enhances remodeling and autoimmunity. In the present chapter, we synthesize the epidemiological and mechanistic evidence linking individual viruses to PAH, map the virus-TLR-vessel interface, and assess translational implications: biomarkers (e.g., IFN-I/IL-6 signatures, circulating mtDNA), risk stratification, and therapeutic targets (i.e., antiviral therapies, TLR modulation, targeting the IL-6 axis, and restoring BMPR2 signaling) [35,37,180,181].

### 3.1. Epstein–Barr Virus (EBV) Infection

Epstein–Barr virus (EBV) is a lymphotropic herpesvirus that persists in the human body in a latent form, undergoing periodic reactivation. Its ability to infect and transform B lymphocytes, together with its capacity to maintain persistent latency, makes it one of the best-characterized oncogenic factors implicated in the pathogenesis of lymphomas and epithelial cancers [182,183,184,185]. Following primary infection of epithelial cells, EBV colonizes B lymphocytes, where its genome remains latent. The IFI16 protein enables the reactivation and long-term persistence of the virus in host cells. Although infection is typically asymptomatic, in immunocompromised individuals it promotes the development of tumors and the deregulation of the immune response [183,186,187].

In the context of PAH, EBV is considered a potential cofactor that initiates or exacerbates vascular remodeling. Viral reactivation leads to the release of viral PAMPs and secondary DAMPs, which activate TLR and inflammatory pathways in the vascular wall. This leads to the chronic activation of NF-κB, increased cytokine production—including IL-6, endothelial dysfunction, endothelial-to-mesenchymal transition (EndMT), the proliferation of PASMCs, and immunothrombotic phenomena, which are processes that lead to increased vascular resistance in the pulmonary circulation [188,189,190,191].

Observations from studies on idiopathic pulmonary fibrosis indicate a significant role of EBV in modulating inflammatory and fibrotic processes. Treatment with Ganciclovir has been shown to attenuate disease progression in patients with advanced disease [192]. Calabrese et al. identified the presence of herpesvirus genomes in 40% of the study population in lung biopsies of patients with idiopathic pulmonary fibrosis, with EBV accounting for 59.1% of the positive cases. The presence of the virus correlated with pulmonary arterial intimal thickening and increased expression of TGF-β, i.e., markers of remodeling and a proinflammatory vascular microarchitecture [193,194]. The collected data suggest that EBV may modulate the phenotype and dynamics of PAH, particularly in patients with concomitant interstitial lung disease or immunosuppression. This requires further translational research focused on:

- Identification of target cells and TLR pathways activated by EBV in the lung.
- Validation of biomarkers, such as plasma EBV DNA and IL-6/TGF-β signatures.
- Assessment of the therapeutic potential of strategies combining antiviral treatment with modulation of the innate immune response and blocking the pro-remodeling cytokine and factor axes.

Therefore, EB appears to be a factor capable of modulating the course of PAH by influencing inflammation and pulmonary vascular remodeling, thereby opening new avenues for diagnosis and treatment [188,189,190,191,192,193,194].

### 3.2. Human Immunodeficiency Virus (HIV) Infection

PAH is a rare (approximately 0.5%) but serious complication of HIV infection [195,196,197]. The association of HIV and the development of pulmonary hypertension was first described in 1987 by Kim and Factor [198]. Given the high and still increasing prevalence of HIV infection—estimated at 40.8 million people worldwide according to WHO data from 2024—HIV may become one of the leading causes of pulmonary arterial hypertension globally [199].

Modern antiretroviral treatment based on HAART (highly active antiretroviral therapy) and the effective treatment of opportunistic infections significantly extend the survival period of HIV-infected patients [200,201,202]. As a result, late complications, including PAH, are being observed with increasing frequency [203]. The pathogenic mechanism underlying the development of pulmonary arterial hypertension in the course of HIV infection remains unclear. HIV and its associated proteins are not present in the vascular endothelium [35,204,205,206]. An indirect role of HIV in the pathogenesis of pulmonary hypertension has been postulated. HIV stimulates monocytes, macrophages, and lymphocytes to secrete proinflammatory cytokines associated with the development of idiopathic pulmonary hypertension: IL-1, IL-6, TNF, and platelet-derived growth factor [35,207,208]. Viral proteins—such as negative factor (Nef), Tat protein, and glycoprotein 120 (gp-120)—can initiate vascular oxidative stress, endothelial damage, and the proliferation and migration of smooth muscle cells, leading to the development of PAH [209,210,211,212].

No correlation has been demonstrated between the severity of pulmonary arterial hypertension and the severity of HIV infection, the degree of immunodeficiency, or the number of CD4+ T lymphocytes [213,214]. In lung biopsies with complex plexiform lesions, the presence of HIV was not detected. This observation suggests that the virus may also exert indirect effects, through the induction of inflammatory process and growth factors, thereby acting as a trigger mechanism in predisposed patients [35,215,216].

### 3.3. Hepatitis C Virus (HCV) Infection

Hepatitis C virus (HCV) belongs to the *Flaviviridae* family and is a small, enveloped, positive-strand RNA virus. In humans, it is responsible for the development of chronic hepatitis C, which in some cases leads to cirrhosis, liver failure, and hepatocellular carcinoma; it is also associated with some lymphomas [217,218,219]. According to data from 2022, HCV infection affects approximately 50 million people worldwide (0.6% of the adult population), with 1.5 million new cases annually. Despite the availability of effective antiviral therapies, mortality rates remain high, primarily due to complications of cirrhosis and HCC [220,221,222,223]. The pathophysiological links between HCV infection and the development of PAH are not clearly defined; however, experimental data indicate the involvement of nonstructural proteins of the virus. The NS5A protein has been shown to activate the STAT3 axis, a key mechanism that promotes pulmonary artery smooth muscle cell (PASMC) proliferation and disrupts endothelial homeostasis. In contrast, other NS3–NS5A/B proteins induce COX-2 expression and increase prostaglandin E_2_ production, thereby fostering a proinflammatory environment conducive to vascular remodeling [224,225,226,227,228,229]. These mechanisms may link chronic HCV infection with the activation of innate inflammatory responses, including the TLR–NF-κB–IL-6 axis, perpetuating the inflammatory-proliferative process in the pulmonary artery wall.

The introduction of direct-acting antiviral drugs (DAAs) has revolutionized the treatment of HCV. An example is sofosbuvir, a nucleotide inhibitor of HCV RNA polymerase, which effectively inhibits viral replication [230]. Interferon-α remains used in some therapeutic regimens. However, clinical reports indicate that both interferon-α and sofosbuvir may induce or exacerbate PAH in some cases. A study by Savale et al. demonstrated that interferon-α-induced PAH has a relatively mild clinical and hemodynamic phenotype, characterized by low right atrial pressure, a moderate increase in pulmonary vascular resistance, normal cardiac output, and only slightly reduced exercise tolerance [227]. Renard et al. described cases of PAH development or progression during sofosbuvir treatment, which was attributed to a rapid decrease in vasodilatory mediators following HCV eradication and secondary activation of the STAT3 axis, potentially unmasking latent or previously stable PAH [224]. HCV infection may therefore promote the development of PAH by activating the STAT3 and COX-2/PGE_2_ pathways, leading to increased PASMC proliferation, endothelial dysfunction, and chronic inflammation. At the same time, selected antiviral therapies may modify the hemodynamic course in specific situations. This requires further translational research aimed at identifying biomarkers predisposing to PAH in HCV, monitoring patients during antiviral treatment, and developing therapeutic strategies that simultaneously control HCV infection and minimize the risk of pulmonary circulation disorders [224,225,226,227,228,229].

### 3.4. Human Endogenous Retrovirus K (HERV-K) Infection

Human endogenous retrovirus K (HERV-K) is one of the most active and conserved members of the HERV family, which are remnants of ancient retroviral infections integrated into the human genome and transmitted vertically through successive generations [231,232,233,234,235]. Although HERVs lack classical replication capacity, they retain the potential for transcription and translation, and their expression can be reactivated by cellular stress, epigenetic disruption, or exogenous infections [236,237,238]. HERV-K has been associated with testicular malignancies, melanoma, and neurodegenerative diseases, among others, indicating its pathogenic potential [239,240,241]. In the context of PAH, the observations of Saito et al. are significant. They demonstrated increased HERV copy numbers, particularly HERV-K, in the lungs of patients with PAH. Analyses also revealed increased expression of transcripts encoding HERV-K dUTPase and HERV-K(II) isoforms in the absence of detectable exogenous virus infection [242]. Mechanistically, recombinant HERV-K dUTPase induced expression of the SAMHD1 protein, which regulates deoxynucleotide metabolism and participates in the antiviral response, in both monocytes and PAECs. This, in turn, resulted in pronounced proinflammatory activation of monocytes, cytokine expression, and endothelial cell apoptosis, directly promoting endothelial dysfunction and initiating vascular remodeling [242].

In animal models, administration of HERV-K dUTPase to rats resulted in the development of a PAH phenotype, characterized by morphological and inflammatory changes in the pulmonary vessels and hemodynamic disturbances, including increased pulmonary arterial pressure [242]. These results suggest that reactivation of endogenous HERV-K sequences may itself generate PAH-like pathology, independent of exogenous virus infection. The collected data indicate that HERV-K may influence the development of PAH through synergistic mechanisms: direct endothelial damage associated with PAEC apoptosis, chronic inflammatory activation of monocytes, and the perpetuation of a proinflammatory-proliferative microenvironment, as well as potential interactions with innate immune sensors such as TLRs and NLRs, which may recognize HERV transcription products as danger signals. This results in increased NF-κB signaling, IL-6 induction, and activation of pathways leading to PASMC proliferation and immunothrombosis. In light of these observations, HERV-K appears not only as a biomarker of active pulmonary vascular remodeling but also as a potential translational target. Directions for further research should include strategies to limit HERV transcription (e.g., epigenetic modulation), neutralization of their protein products, or inhibition of secondary inflammatory pathways induced by HERV-K reactivation. Although knowledge in this area is still limited, available evidence indicates that HERV-K may be an important endogenous factor modulating the pathophysiology of PAH [243,244,245,246,247].

### 3.5. SARS-CoV-2 Infection

The COVID-19 pandemic caused by SARS-CoV-2 has compounded the already known susceptibility of the pulmonary vascular bed to inflammatory and thrombotic injuries and revealed a particular vulnerability of patients with previously diagnosed PAH/PH. This population has been reported to exhibit a higher risk of hospitalization, right ventricular decompensation, and death, with in-hospital mortality rates reaching 25–43% in selected registries [247,248,249,250,251,252,253,254]. Increasing evidence also points to persistent vascular sequelae following COVID-19, including secondary forms of pulmonary hypertension in convalescent patients, especially after severe pneumonia or ARDS [255,256,257,258]. At a mechanistic level, SARS-CoV-2 engages innate immune pathways relevant to pulmonary vascular remodeling: viral structural proteins (E and—in specific experimental systems—S) activate TLR2, while S can also trigger TLR4 in a context-dependent manner; in parallel, viral RNA activates endosomal TLR3/7/8. These inputs converge on MyD88→NF-κB and TRIF→IRF3/7 cascades, driving IL-6/TNF and type-I interferon responses, oxidative stress, and secondary HIF-1α activation—signaling that sustains a pro-inflammatory/pro-proliferative vascular phenotype [259,260,261,262]. SARS-CoV-2–induced endothelial dysfunction is well documented: endotheliitis, barrier disruption, immunothrombosis, and angiogenesis culminate in thrombotic microangiopathy and increased right-ventricular afterload; ACE2 down-regulation further contributes to RAAS imbalance, vasoconstriction, and capillary leak—hallmarks overlapping with PH/PAH pathogenesis and initiating EndMT-driven intimal/adventitial remodeling [263,264]. Notably, despite this overlap, direct experimental proof that SARS-CoV-2-evoked TLR signaling causally perturbs canonical PAH pathways (e.g., BMPR2) remains limited; conversely, TLR3 activation can upregulate BMPR2 and ameliorate experimental PH, whereas TLR4 is implicated in pulmonary vascular homeostasis and its loss favors remodeling—underscoring context dependence [19,22].

The basis for these phenomena is extensive damage to the pulmonary endothelium, accompanied by endotheliitis, immunothrombosis, and vascular wall remodeling—processes crucial for the pathogenesis of PH/PAH [188,265,266,267]. SARS-CoV-2 enters cells via ACE2, which promotes dysregulation of the RAAS axis (ACE2 deficiency and relative predominance of angiotensin II), vasoconstriction, capillary leakage, and a prothrombotic state [268,269,270]. In parallel, thrombotic microangiopathy develops, characterized by generalized microvascular thrombosis, which impairs perfusion and increases right ventricular afterload [271,272,273,274]. Histopathological and imaging studies confirm generalized endothelial activation, inflammatory infiltrates, and vascular barrier damage, which initiate EndMT and remodeling of the intima and adventitia [275,276,277,278]. The molecular mechanisms involve innate immune pathways (TLR activation by viral PAMPs and secondary DAMPs), NF-κB/AP-1 cascades with IL-6/TNF-α production, oxidative stress, and the secondary activation of HIF-1α—a constellation of signals that perpetuates the proinflammatory and proliferative phenotype of the pulmonary vasculature [260,279,280,281,282].

The acute phase may result in the development or exacerbation of chronic PH. Phenotypes secondary to interstitial lung disease and post-ARDS fibrosis, as well as forms associated with chronic hypoxemia and persistent impairment of vascular reserve, are observed after COVID-19. In some echocardiographic series, features of PH were detected in up to 70% of patients several months after recovery, particularly in older individuals with obesity and prolonged desaturation [257,282,283,284]. In patients with baseline PAH/CTEPH, SARS-CoV-2 infection more often leads to acute right ventricular failure and worse short-term outcomes, reflecting the cumulative effects of microthrombosis, vasoconstriction, and increased pulmonary resistance [285,286,287,288,289].

From a therapeutic perspective, continued PAH-specific therapy (endothelin receptor antagonists, PDE-5 inhibitors, analogs/prostanoids) is recommended during the acute phase of COVID-19 to reduce the risk of decompensation. Inpatient management should be individualized and include oxygen therapy, diuretic therapy, and hemodynamic support according to the patient’s profile [290,291,292]. The issue of reinitiating or intensifying PAH-specific therapy after infection remains ambiguous. It requires evaluation in prospective studies, as do algorithms for monitoring convalescent patients for the development of new-onset PH (biomarkers of RV overload, echocardiogram, and eligibility for hemodynamic testing) [293,294]. In summary, SARS-CoV-2 represents both an acute stressor on the lung endothelium and a potential long-term driver of pulmonary vascular disease; mechanistic, translational, and clinical studies are needed to precisely define the risk trajectory and optimize therapeutic and surveillance strategies in this vulnerable population.

## 4. Conclusions

The collected evidence indicates that the balance of TLRs largely determines the course and prognosis of PAH—specifically, the balance between the proinflammatory MyD88–NF-κB/IL-6 axis and the protective TRIF–IFN–α axis. Activation of most receptors (TLR1/2, 4, 5/6, 7/8, 9) amplifies inflammation, oxidative stress, immunothrombosis, and vascular wall remodeling, thereby increasing pulmonary vascular resistance. In contrast, TLR3 exerts an opposing effect by stabilizing the endothelium and inhibiting remodeling, and its dysfunction is associated with more severe disease. Viral infections and reactivations (HIV-1, EBV, HCV, HERV-K, SARS-CoV-2) further tilt this balance toward pro-remodeling cascades in genetically or immunologically predisposed individuals. Taken together, these observations suggest that more effective therapeutic strategies should go beyond vasodilation alone and target modulation of the innate immunity of the vascular wall—enhancing TLR3/TRIF signaling and selectively suppressing the overactive MyD88–NF-κB/IL-6 axis (including through IL-6 modulation), combined with antiviral therapy if necessary. Precise patient selection based on simple, clinically available indicators (e.g., IFN-I/IL-6 signatures or markers of mitochondrial damage) and methodological caution will be crucial, as non-selective TLR blockade may carry a risk of infection. This therapeutic approach, based on TLR and viral biology, offers a real chance to slow vascular remodeling and improve PAH treatment outcomes.

## Figures and Tables

**Table 2:** Comparison of the role of TLR1 and TLR2 in the regulation of inflammatory response, vascular remodeling, and hemodynamics in the pathogenesis of pulmonary arterial hypertension (PAH) (based on [39,40,41,42,43,44,45,46,47,48,49,50,51,52,53]).

Aspect	TLR1	TLR2
Regulation/expression in PAH (pre-clinical models)	↓ Expression in the lungs of rats with MT-PAH; positive correlation with neutrophil and monocyte counts, negative correlation with resting CD4+ lymphocytes	↑ Lung expression in MT-PAH; macrophage infiltration, activation of the TLR–RIPK3 axis, and dysregulation of TLR/NLR pathways promote progression of vascular remodeling
Compartments/target cells (inference from data)	Modulation of the innate component (neutrophils, monocytes) and, indirectly, T lymphocytes; role in sensing pathogenic stimuli	Endothelial cells (EC), pulmonary artery smooth muscle (PASMC), fibroblasts, leukocytes; high activity in the pulmonary vascular microenvironment
Signal paths	Mainly MyD88 → NF-κB, induction of inflammatory mediators (inferred from TLR1/2 function)	MyD88 → NF-κB; induction of IL-1, IL-6, TNF-α; association with RIPK3 and interactions with NLRs
Impact on key mechanisms for PAH	Indirectly enhancing innate effector cell recruitment; potentially driving neutrophil/monocyte-dependent inflammatory responses	↓ PASMC apoptosis; ↑ PASMC proliferation; ↑ TGF-β/PDGF; fibroblast activation and ECM deposition; ↑ oxidative stress; ↑ ICAM-1/VCAM-1 (facilitated leukocyte migration); EC dysfunction (permeability, ↓ NO)
Vascular/hemodynamic consequences	Potential involvement in the initiation and maintenance of inflammation affecting vascular remodeling	Increased vascular remodeling and inflammation; correlation with severity and deterioration of right ventricular function in animal models
Biomarkers and therapeutic implications	Candidate immunoinfiltration biomarker (requires translational validation)	Potential biomarker of inflammatory activity; modification of TLR2 activity considered a therapeutic target in PAH

Abbreviations: PAH—Pulmonary arterial hypertension, MT-PAH—Monocrotaline-induced pulmonary arterial hypertension (preclinical rat model), RIPK3—Receptor-interacting protein kinase 3, NLR—NOD-like receptor, EC—Endothelial cells, PASMC—Pulmonary artery smooth muscle cells, MyD88—Myeloid differentiation primary response 88, NF-κB—Nuclear factor kappa-light-chain-enhancer of activated B cells, IL-1—Interleukin-1, IL-6—Interleukin-6, TNF-α—Tumor necrosis factor-alpha, TGF-β—Transforming growth factor-beta, PDGF—Platelet-derived growth factor, ECM—Extracellular matrix, ICAM-1—Intercellular adhesion molecule-1, VCAM-1—Vascular cell adhesion molecule-1, NO—Nitric oxide; ↑ means increase; ↓ means decrease.

**Table 3:** Comparative aspects of TLR5 and TLR6 in PAH (based on [15,42,97,98,99,100,101,102,103,104,105,106,107,108,109,110,111,112,113,114,115,116,117,118,119,120]).

Aspect	TLR5	TLR6 (with TLR2)
Status of evidence in PAH	No dedicated functional studies have been conducted in PAH models; however, transcriptomic analyses indicate a significant induction of TLR5 in the lungs of patients and mice with PH.	No direct clinical/animal studies in PAH; available RNA-seq analyses and data from stimulation of the TLR2/6 heterodimer in PASMCs.
Ligand(s)/recognition	Bacterial flagellin.	The TLR2/6 heterodimer recognizes diacylated bacterial lipopeptides (e.g., MALP-2).
Canonical signaling pathway	MyD88 → IRAK → NF-κB → TNF-α, IL-6, IL-8.	MyD88 → NF-κB (via TLR2/6 heterodimer) → IL-1β, IL-6, TNF-α.
Cellular/vascular effects (inferred)	Induction of inflammation similar to TLR2/TLR4: leukocyte recruitment, oxidative stress, vascular wall remodeling.	Promotion of proliferation and vascular remodeling, an increase in endothelin-1, and persistence of inflammation in the pulmonary microenvironment.
Angiogenesis/repair	No data available.	The TLR2/6 complex enhances angiogenesis (via GM-CSF) both in vitro and in vivo, supporting blood flow, immune cell recruitment, and tissue regeneration (e.g., in the liver).
Genetic associations	No reports in the cited material.	TLR6 A359T>C: predisposition to Legionnaires’ disease; Ser249Pro: possible association with asthma in selected populations.
Implications for PAH	Potential pro-inflammatory and pro-remodeling role; requires verification in in vivo/in vitro studies.	Potential therapeutic target: antagonism of signaling via the TLR2/6 → NF-κB axis to reduce inflammation and remodeling.

Abbreviations: PAH—Pulmonary arterial hypertension, PH—Pulmonary hypertension, RNA-seq—RNA sequencing, PASMC—Pulmonary artery smooth muscle cells, MyD88—Myeloid differentiation primary response 88, IRAK—Interleukin-1 receptor–associated kinase, NF-κB—Nuclear factor kappa-light-chain-enhancer of activated B cells, TNF-α—Tumor necrosis factor alpha, IL-6—Interleukin-6, IL-8—Interleukin-8, IL-1β—Interleukin-1 beta, Endothelin-1—Endothelin-1, GM-CSF—Granulocyte-macrophage colony-stimulating factor, MALP-2—Macrophage-activating lipopeptide-2, → denotes a relationship, a causal connection, or a transition to the next piece of information.

## Data Availability

This article is a review and does not involve the generation or analysis of new primary data. All data supporting the statements and conclusions of this work are contained within the manuscript.

## Abbreviations

The following abbreviations are used in this manuscript:

**Abbreviation**	**Expansion**
ACE2	angiotensin-converting enzyme 2
ANA	antinuclear antibodies
AP-1	Activator Protein-1
ARDS	acute respiratory distress syndrome
BMPR2	bone morphogenetic protein receptor type 2
CD281–CD290	CD designations corresponding to TLR1–TLR10
CNS	central nervous system
COX-2	cyclooxygenase-2
CpG DNA	unmethylated CpG motif–containing DNA
CpG ODN	CpG oligodeoxynucleotide
CRRT	continuous renal replacement therapy
CTEPH	chronic thromboembolic pulmonary hypertension
DAA	direct-acting antiviral
DAMP	damage-associated molecular pattern
dsDNA	double-stranded DNA
dsRNA	double-stranded RNA
EBV	Epstein–Barr virus
EC	endothelial cell(s)
ECM	extracellular matrix
ECMO	extracorporeal membrane oxygenation
E6446	selective TLR9 antagonist
EndMT	endothelial-to-mesenchymal transition
ET-1	endothelin-1
GM-CSF	granulocyte–macrophage colony-stimulating factor
gp120	HIV-1 envelope glycoprotein 120
gp96	endoplasmin/GRP94 (heat-shock protein HSP90B1)
HAART	highly active antiretroviral therapy
Hb	hemoglobin
HCC	hepatocellular carcinoma
HCV	hepatitis C virus
HERV-K	human endogenous retrovirus K
HIF-1α	hypoxia-inducible factor-1 alpha
HIV-1	human immunodeficiency virus type 1
HMGB1	high-mobility group box 1
HSP	heat-shock protein
Hsp70	heat-shock protein 70
ICAM-1	intercellular adhesion molecule-1
Id1	inhibitor of DNA binding 1
IFN	interferon (general)
IFN-I	type I interferons
IFN-α	interferon-alpha
IFN-β	interferon-beta
IFN-γ	interferon-gamma
IFNA1	interferon alpha 1 gene
IFNB1	interferon beta 1 gene
IL-1	interleukin-1
IL-6	interleukin-6
IL-8	interleukin-8
IRAK	interleukin-1 receptor–associated kinase
IRF3	interferon regulatory factor 3
LDH	lactate dehydrogenase
LL-37	human cathelicidin antimicrobial peptide
LPS	lipopolysaccharide
MALP-2	macrophage-activating lipopeptide-2 (TLR2/6 agonist)
miRNA	microRNA
MRI	magnetic resonance imaging
mtDNA	mitochondrial DNA
MyD88	myeloid differentiation primary response 88
NOX (NADPH oxidase)	nicotinamide adenine dinucleotide phosphate oxidase system
NETs	neutrophil extracellular traps
NF-κB	nuclear factor kappa-B
NLR	NOD-like receptor
NO	nitric oxide
Nox1	NOX family isoform 1
Nox4	NOX family isoform 4
NS3	nonstructural protein 3 (HCV)
NS5A	nonstructural protein 5A (HCV)
NS5B	nonstructural protein 5B (HCV)
Nef	HIV-1 negative factor protein
PAMP	pathogen-associated molecular pattern
PAEC	pulmonary artery endothelial cell(s)
PAH	pulmonary arterial hypertension
PASMC	pulmonary arterial smooth muscle cell(s)
PDE-5	phosphodiesterase type 5
PDGF	platelet-derived growth factor
PGE_2_	prostaglandin E_2_
PH	pulmonary hypertension
poly(I:C)	polyinosinic:polycytidylic acid (synthetic dsRNA; TLR3 agonist)
p53	tumor protein p53
pDC	plasmacytoid dendritic cell(s)
RAAS	renin–angiotensin–aldosterone system
R848	resiquimod (TLR7/8 agonist)
R840	imidazoquinoline TLR7/8 agonist (experimental)
RANTES (CCL5)	Regulated upon Activation, Normal T-cell Expressed and Secreted
RIPK3	receptor-interacting protein kinase 3
RNA-seq	RNA sequencing
ROS	reactive oxygen species
RV	right ventricle
RVEF	right ventricular ejection fraction
RVSP	right ventricular systolic pressure
SARS-CoV-2	severe acute respiratory syndrome coronavirus 2
SLE	systemic lupus erythematosus
Smad1/5/8	SMAD proteins downstream of BMP/TGF-β signaling
SMC	smooth muscle cell(s)
ssRNA	single-stranded RNA
STAT3	signal transducer and activator of transcription 3
SSc	systemic sclerosis
SU5416	VEGFR inhibitor used to induce experimental PAH
Tat	HIV trans-activator of transcription protein
TGF-β	transforming growth factor-beta
Th17	T helper 17 cells
TIR	Toll/Interleukin-1 receptor (domain)
TLR	Toll-like receptor(s)
TMAO	trimethylamine-N-oxide
TNF	tumor necrosis factor
TNF-α	tumor necrosis factor-alpha
TPVR	total pulmonary vascular resistance (index)
TRIF	TIR-domain–containing adapter-inducing interferon-β
Treg	regulatory T cell(s)
TXA_2_	thromboxane A_2_
VCAM-1	vascular cell adhesion molecule-1
VEGF	vascular endothelial growth factor
VEGFR	vascular endothelial growth factor receptor
WHO	World Health Organization
mPAP	mean pulmonary arterial pressure
